# Efficient adaptive feature aggregation network for low-light image enhancement

**DOI:** 10.1371/journal.pone.0272398

**Published:** 2022-08-23

**Authors:** Canlin Li, Pengcheng Gao, Jinhua Liu, Shun Song, Lihua Bi

**Affiliations:** 1 School of Computer and Communication Engineering, Zhengzhou University of Light Industry, Zhengzhou, China; 2 Shanghai Film Academy, Shanghai University, Shanghai, China; University of Engineering & Technology, Taxila, PAKISTAN

## Abstract

Existing learning-based methods for low-light image enhancement contain a large number of redundant features, the enhanced images lack detail and have strong noises. Some methods try to combine the pyramid structure to learn features from coarse to fine, but the inconsistency of the pyramid structure leads to luminance, color and texture deviations in the enhanced images. In addition, these methods are usually computationally complex and require high computational resource requirements. In this paper, we propose an efficient adaptive feature aggregation network (EAANet) for low-light image enhancement. Our model adopts a pyramid structure and includes multiple multi-scale feature aggregation block (MFAB) and one adaptive feature aggregation block (AFAB). MFAB is proposed to be embedded into each layer of the pyramid structure to fully extract features and reduce redundant features, while the AFAB is proposed for overcome the inconsistency of the pyramid structure. EAANet is very lightweight, with low device requirements and a quick running time. We conducted an extensive comparison with some state-of-the-art methods in terms of PSNR, SSIM, parameters, computations and running time on LOL and MIT5K datasets, and the experiments show that the proposed method has significant advantages in terms of comprehensive performance. The proposed method reconstructs images with richer color and texture, and the noises is effectively suppressed.

## 1 Introduction

In low-light conditions, the captured image suffers from texture and color deviations and contains a lot of noises, which affects the visual perception. Low-light environment also increase the difficulty of high-level vision tasks, such as image detection [1, 2] and visual tracking [3, 4]. Luminance variation is one of the main factors that affect the performance of such task. One solution is to use a capture device with high international standards organization (ISO) to obtain high quality images, however, this method is insufficient and expensive for image improvement. Therefore, image enhancement techniques are often used to enhance low-light images. Researchers have proposed a large number of low-light image enhancement methods, which can be classified into three categories: Histogram equalization-based, Retinex-based, and learning-based.

Histogram equalization is a classical method that enhances the contrast of the whole image by extending the dynamic range of the pixels. The method is simple and convenient, but the enhanced image lacks details. Some improved methods based on HE [5, 6] further improve the visual perception of the image, but still cannot meet the practical demands.

Some works [7–11] combine Retinex theory to decompose the image into a illumination map and a reflection map. The enhanced image is obtained by fusing the processed illumination map and reflection map. However, it is difficult to estimate the illumination map of the image accurately by these methods, because the image has different degrees of degradation in low-light environment. The enhanced images usually have low contrast, blurred details and contains strong noises.

Many learning-based methods for low-light image enhancement have been proposed. For example, some end-to-end methods [12–14] design complex structures to integrate features, but the generated images lack texture and contain noises. Some methods [15, 16] enhance low-light images with the help of physically interpretable Retinex theory. These methods achieve better results than traditional methods but the suppression of noises is inadequate. There are also unsupervised methods [17, 18] that alleviate the need for paired datasets and improve the generalization ability of the methods, but these methods are difficult to train and the generated images have color deviations and noises.

Existing learning-based methods are inefficient in the utilization of features, and the reconstructed images lack detail and have noises. Some methods try to combine pyramid structures to learn features, using a coarse-to-fine strategy to capture fine image details, but the inconsistency of pyramid structures leads to insufficient communication between deep semantic information and shallow fine-grained information. Meanwhile, these methods are complex and require a lot of device resources, which are difficult to be applied to mobile devices or to assist high-level vision tasks. To address these problems, we design an efficient adaptive feature aggregation network (EAANet) for low-light image enhancement. Specifically, we propose the multi-scale feature aggregation block (MFAB) and the adaptive feature aggregation block (AFAB). MFAB contains information aggregation block (IAB) and Dual Attention Block (DAB), during the inference phase, the asymmetric convolution of IAB is fused into a standard convolution, which can enhance the feature representation without increasing the number of parameters, DAB refines the features from both spatial and channel aspects to reduce the feature redundancy. In order to improve the feature scale inconsistency of the pyramid structure, AFAB adaptively selects features from the pyramid structure to establish a connection between the semantic information at the deep layer and the fine-grained information at the shallow layer. In addition, both blocks are lightweight. Through the collaboration in the two blocks, the proposed method reconstructs images with good luminance, color and texture.

The main contributions of this paper are as follows:

1. We propose an efficient adaptive feature aggregation network (EAANet) for low-light image enhancement. Extensive experiments show that the proposed method can effectively reconstruct the color and texture of the image and suppress the image noises.
2. We propose a multi-scale feature aggregation block (MFAB), which improves feature representation by asymmetric convolution and reduces redundant features using a dual attention mechanism. MFAB is a lightweight block and it’s very efficient. In the inference stage, the asymmetric convolution is fused into a standard convolution to further reduce the parameters and computations.
3. In order to address the inconsistency of the pyramid structure, we propose an adaptive feature aggregation block (AFAB). The block enhances the interaction between the shallow fine-grained information and the deep semantic information of the pyramid to improve the texture and color of the image.
  The remainder of this paper is organized as follows. We introduce the related work on low-light image enhancement methods in Section 2. Our proposed EAANet is described in detail in Section 3. We give the experiments and the comparison of results in Section 4. Section 5 is the ablation study of EAANet. Section 6 is the conclusion.

## 2 Related work

In this section, we introduce the work related to low-light image enhancement methods, including histogram equalization-based methods, Retinex-based methods, and learning-based methods.

### 2.1 Histogram equalization-based methods

Inspired by Histogram equalization (HE), a large number of improved methods based on HE have been proposed. For example, Pizer et al. [5] proposed an contrast limited adaptive histogram equalization (CLAHE), which restricts each sub-histogram. CLAHE improves the image contrast and suppresses noises, but it first divides the image into multiple block regions, which result in block artifacts in the reconstructed image. To solve the block artifact phenomenon, Srinivasan et al. [19] proposed a histogram equalization using local region stretching, which divides the image into multiple areas according to the gray level, and then histogram equalization is performed for each gray level area. Ibrahim et al. [6] proposed dynamic histogram equalization with preserved luminance, which has a good effect on some locally underexposed images by calculating the global average luminance. However, this method is greatly limited when the image luminance is extremely dark. Although histogram equalization-based methods and their improvement methods are simple and convenient, they usually suffer from image distortion and noises problems.

### 2.2 Retinex-based methods

Retinex is a theory that imitates human vision, and it is widely used in image enhancement tasks. In recent years, a large number of Retinex-based methods have been proposed. For example, SSR [7], MSR [8] and MSRCR [9], they are pioneering methods. Wang et al. [10] proposed a method to improve the contrast and naturalness of images, but the method still produces severe noises. Cai et al. [11] proposed joint intrinsic-extrinsic prior model (JieP) to protect structural integrity through shape prior. Guo et al. [20] proposed LIME to accelerate the optimization of the illumination map, which greatly reduces the computations. Xu et al. [21] proposed a new detail-preserving variational enhancement model that uses the l1 prior to constraining the illumination map. Retinex-based methods are usually better for image color fidelity, but these methods are poorly adaptable and also prone to artifacts, strong noises, and overexposure. In addition, it is difficult to estimate the illumination map of extremely low-light images with heavy noises.

### 2.3 Learning-based methods

In recent years, learning-based methods achieved great progress. LLNet [12] was proposed as an early end-to-end method for low-light image enhancement. Wang et al. [13] proposed a globally aware network (GLAD), which incorporates a pyramid structure to extract image features. MIRNet [14] combines a residual structure and an attention mechanism to reconstruct the correct luminance and color. Some methods combine Retinex theory, for example. Wei et al. [15] proposed RetinexNet, which designed a decomposition net and an enhancement net to decompose and process images. Inspired by Ref [15], Zhang et al. [16] proposed KinD, which designed a light adjustment block to flexibly adjust the luminance. Wang et al. [22] proposed DeepUPE, which combines Retinex and bilateral filtering. Liu et al. [23] proposed RAUNA, which designed a decomposition network with explicit and implicit priors, and taken into account both global and local brightness in the augmented network. Some methods try to learn enhancement results from unpaired data, such as Jiang et al. [24] proposed a degradation-to-refinement generation network (DRGN). Li et al. [17] proposed zero-reference depth curve estimation (Zero-DCE++) for low light image enhancement. Risheng et al. [18] proposed Retinex-inspired unrolling with cooperative prior architecture search (RUAS), which first searches the network architecture through the training set, and then finds the low-light prior structure from the compact search space. These methods further liberate the restriction on the dataset, but they are not stable in training. Compared with traditional methods, learning-based methid combined with Retinex theory can better estimate the illumination and reflection maps and have better enhancement performance, but the noises suppression of these methods is still inadequate. Compared with depth Retinex-based methods, the end-to-end methods are more effective in suppressing noises. The end-to-end methods usually have low complexity and has great potential for assisting high-level vision tasks and applying on mobile devices. Recently, Li et al. [25] proposed a luminance-aware pyramid network (LPNet). LPNet is a lightweight end-to-end method, and it has strong competitiveness. LPNet designed a multi-scale contrast feature block for feature extraction and a luminance loss for guiding the model to learn luminance information. However, LPNet uses too many split operations, resulting in insufficient information exchange between channels and the reconstructed image lacks texture. In addition, due to the inconsistency of pyramid structure, the shallow features and deep features of the pyramid structure cannot be fully utilized. This causes color deviations in the reconstructed images by LPNet.

In our work, we propose an efficient adaptive feature aggregation network (EAANet). EAANet contains two important blocks, MFAB and AFAB. MFAB utilizes asymmetric convolution and dual attention mechanisms to fully extract features and capture critical features. Compared with LPNet, MFAB is more efficient to extract features. AFAB overcomes the inconsistency of pyramid structure and enhances the communication between shallow fine-grained information and deep semantic information. We describe the proposed EAANet in Section 3.

## 3 Method

**Fig 1:**
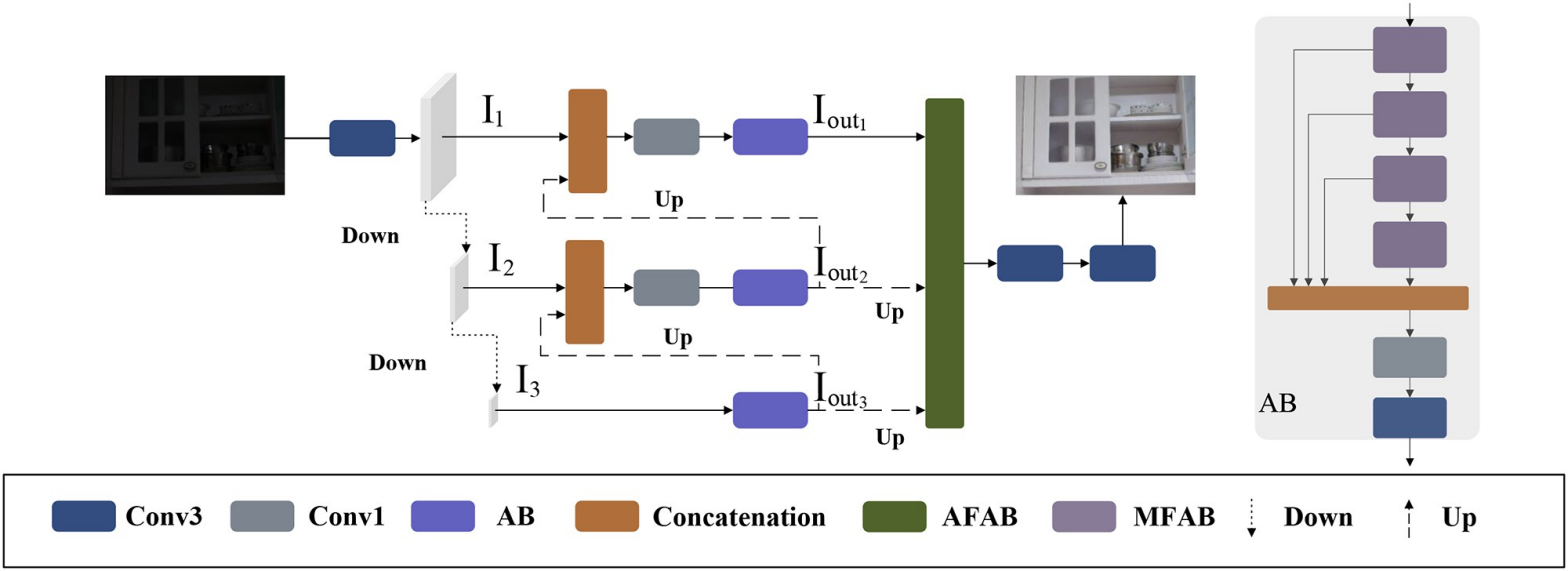
The structure of our proposed EAANet.

In this section, we describe the proposed EAANet. EAANet use a pyramid structure. Pyramid structure is a common multi-scale structure, and it is one of the main methods for extracting multi-scale information. It is widely used in traditional methods [26], convolutional neural network (CNN) based methods [27–30] and Transformer methods [31]. Pyramid structure has a large perceptual field and can provide rich feature information. EAANet has three layers, each layer contains multiple MFABs for extracting features, and the output of each layer is incorporated into the shallow layer to guide the shallow layer to learn more refined features. In addition, the output of each layer is also fed into AFAB to establish connections between the features of different layers. As shown in Fig 1, the input image is first mapped as high-dimensional feature through a convolutional layer and then successively downsampled to obtain features at different scales, the operation can be defined as Eqs (1) and (2):

$$\begin{array}{c} I_{1}=Conv3\left(I_{input}\right), \end{array}$$ (1)

$$\begin{array}{c} I_{n}=F_{down}\left(I_{n-1}\right),n=2,3 \end{array}$$ (2)

where *I*_*input*_ is the low-light image and *I*_*n*_ denotes the corresponding input of the nth layer. *F*_*down*_ denotes a down-sampling. *Conv*3 denotes a 3 × 3 convolutional layer. Some critical features will gradually lose with the increase of network depth. Inspired by Ref [32, 33], we construct an aggregation block (AB) to address the problem of losing critical features with the increasing depth of the network. Each AB contains n multi-scale feature aggregation blocks (MFABs), and the output of each MFAB is concatenated to make full use of the features. We set n to be 4. To avoid generating a large number of parameters, the concatenated features are first passed through a 1 × 1 convolution layer to adjust the number of channels, and then the features are reconstructed by a 3 × 3 convolutional layer. At the bottom layer, the features first pass through an AB, then the outputs of AB are up-sampled and concatenated with the middle layer features obtained from the previous down-sampling operation. Afterwards, the number of channels is adjusted by 1 × 1 convolutional layer and passed again through the AB. By repeating this operation, the output of the AB for each layer can be obtained, this operation can be defined as Eqs (3)–(5):

$$\begin{array}{c} F_{AB}=Conv3\left(Conv1\left(∥MFAB_{out_{1}},MFAB_{out_{2}},...,MFAB_{out_{n}}∥\right)\right) \end{array}$$ (3)

$$\begin{array}{c} I_{out_{3}}=F_{AB}\left(I_{3}\right) \end{array}$$ (4)

$$\begin{array}{c} I_{out_{n}}=F_{AB}\left(Conv1\left(∥F_{up}\left(I_{out_{n+1}}\right),I_{n}∥\right)\right),n=1,2 \end{array}$$ (5)

where $I_{out_{n}}$ is the output of the nth layer. ‖‖ is concatenation operation. *Conv*1 denotes a 1 × 1 convolutional layer. *F*_*AB*_ denotes the corresponding operation of AB. *F*_*up*_ denotes a up-sampling. $MFAB_{out_{n}}$ denotes the output of the i-th MFAB. We describe MFAB in detail in section 3.1. The output of the three layers is fed to AFAB to strengthen the connection between the shallow fine-grained information and the deep semantic information. This operation can be defined as Eq (6):

$$\begin{array}{c} I_{output}=Conv3\left(Conv3\left(F_{AFAB}\left(I_{out_{1}},I_{out_{2}},I_{out_{3}}\right)\right)\right) \end{array}$$ (6)

where *I*_*output*_ is enhanced image. *F*_*AFAB*_ denotes the corresponding operation of AFAB. We describe MFAB in detail in section 3.2. The output of AFAB is passed through two 3 × 3 convolutional layers to obtain the final enhanced image. Our method is end-to-end, so that the whole operation can be defined as Eq (7):

$$\begin{array}{c} I_{output}=F_{EAANet}\left(I_{input}\right) \end{array}$$ (7)

where *F*_*EAANet*_ is the corresponding operation of EAANet. Each down-sampling contains a 2-step 3 × 3 convolution and a 1-step 3 × 3 convolution. Each up-sampling contains bilinear interpolation and a 1-step 3 × 3 convolution. Subsequently, we describe each block of the model and the loss function in details.

### 3.1 Multi-scale feature aggregation block (MFAB)

**Fig 2:**
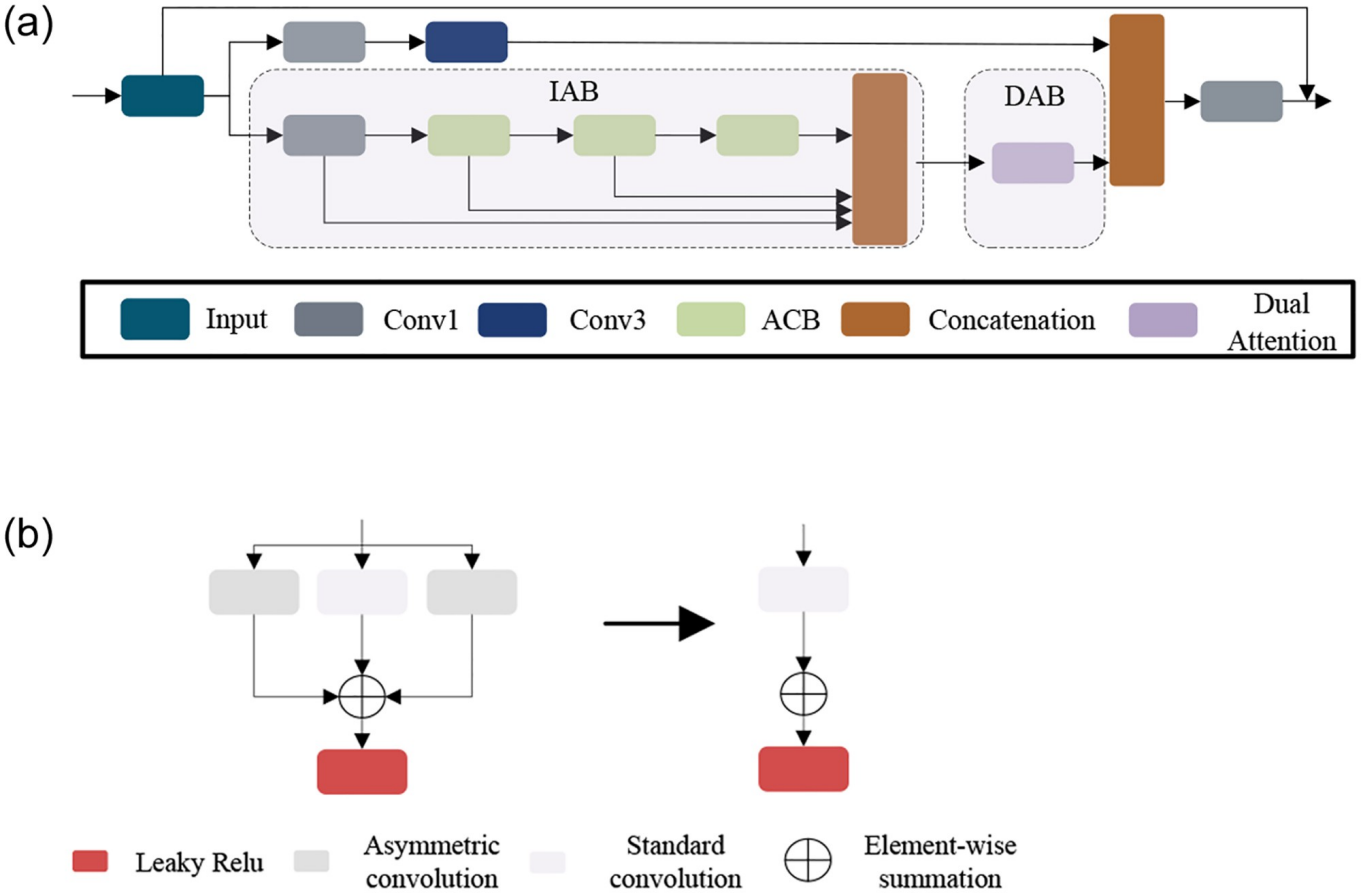
(a) The structure of our proposed MFAB. MFAB contains IAB and DAB. (b) The structure of ACB. ACB transforms to standard convolution in the inference stage.

Some methods designed novel structures to extract abundant features. For example, MSRN [32] uses convolutional layers with different kernels. LPNet [25] splits features along the channel dimension and then passes through different numbers of convolutional layers, respectively. However, the feature extraction capability of these methods is inefficient. Convolutional layer with large kernel generates a large number of parameters, and the split operation can hinder the information interaction between channels and degrade the feature representation. We proposed the MFAB to better extract and utilize the features. As shown in Fig 2, the feature from the previous layer is divided into two branches by two 1 × 1 convolutional layer, respectively. Each branch has only half the number of channels. One branch passes through a 3 × 3 convolutional layer, and then waits for a concatenation with another branch. The other branch can be roughly divided into two parts: An information aggregation block (IAB) and a double attention block (DAB).

Learning-based methods are often necessary to design complex structures to obtain rich feature information. ACNet proposes an efficient method for capturing feature information, it try to enhances the standard convolution kernel using asymmetric convolution. Inspired by ACNet [34], we designed an asymmetric convolution block (ACB), which contains 2 asymmetric convolutions and 1 standard convolution. The outputs of the asymmetric convolution and the standard convolution are combined by an element summing operation and Leaky Relu activation is added. For the IAB, the features pass through three ACB. During the training phase, asymmetric convolution block contains asymmetric convolution and standard convolution, the expression can be defined as Eq (8):

$$\begin{array}{c} F_{ACB}=\delta \left(\left(U*K^{3\times 1}+U*K^{3\times 3}+U*K^{1\times 3}\right)\right) \end{array}$$ (8)

where *U* is the feature of the previous layer. *K* is convolution kernel. *δ* is Leaky Relu activation. During the inference phase, we transform the asymmetric convolution to standard convolution to reduce the number of parameters of the network while maintaining performance, and the operation can be defined as Eq (9):

$$\begin{array}{c} U*K^{3\times 1}+U*K^{3\times 3}+U*K^{1\times 3}=U*\left(K^{3\times 1}⊕K^{3\times 3}⊕K^{1\times 3}\right) \end{array}$$ (9)

where ⊕ is the element-wise addition of the kernel parameters. Receptive field has a great influence on feature extraction, and the receptive fields of the feature output by each ACB are different. We concatenate the output of each asymmetric convolution block to fuse the feature information of different receptive fields.

**Fig 3:**
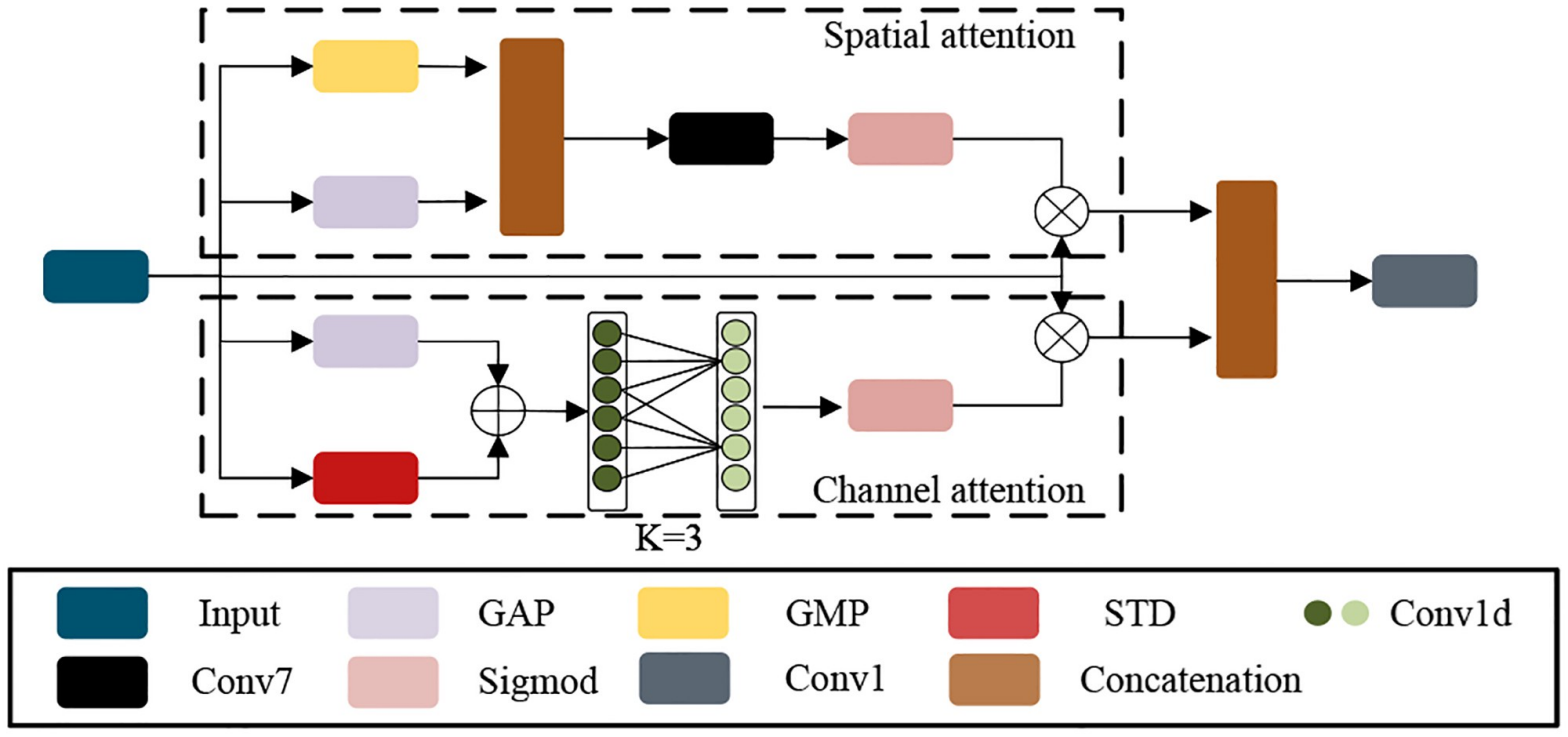
The structure of our proposed dual attention. Conv1d is a 1-Dimension convolution. We set the kernel of Conv1d as 3. ⊗ is element-wise multiplication operation.

Low-light images have a lot of noises and the extracted features are often redundant. Attention mechanisms are used for high-level vision tasks such as image classification, image recognition, etc. It can promote models to focus more on valuable features and fade out useless features. Several methods [25, 35, 36] have demonstrated that the attention mechanism helps low-level visual models to learn the correct texture and suppress noises. Therefore, we try to use the attention mechanism to reduce feature redundancy. Specifically, we designed parallel spatial and channel attention mechanisms. Color degradation is a challenge for low-light image enhancement. Inspired by Ref [37], we added standard deviation (STD) based on global average pooling to the channel attention branch, as shown in Fig 3. The standard deviation is calculated as the degree of dispersion of individual pixels from the mean value of the image. By adding standard deviation, the model can be promoted to pay more attention to edge information, so the color of images can be improved. For the standard deviation, we define *X* = [*x*_1_, *x*_2_, …., *x*_*c*_] as input, whose shape is *C* × *H* × *W*, and the expression can be defined as Eq (10):

$$\begin{array}{c} \varepsilon_{c}=\sqrt{\frac{1}{HW}\sum_{i,j\in x_{c}}^{}{\left(x_{c}^{i,j}-\frac{1}{HW}\sum_{i,j\in x_{c}}x_{c}^{i,j}\right)}^{2}}, \end{array}$$ (10)

where *ε*_*c*_ is the standard deviation of the c-th element of the output. STD can improves the color of images. In order to reduce the computational complexity, existing channel attention mechanisms usually perform channel dimensionality reduction, and this operation lead to the result that the model cannot effectively capture dependencies between channels and lose some important contextual information. Inspired by Ref [38], we use 1-dimensional convolution to avoid dimensional reduction. The output of the global average pool is first element-wise summed with *ε*_*c*_, and then a one-dimensional convolution is used to avoid channel reduction, and the one-dimensional convolution allows sufficient information interaction between channels. The features are normalized by the Sigmoid activation function and finally the original features are rescaled by the element multiplication operation. In the spatial attention branch, we do average pooling and maximum pooling for the spatial dimensions, respectively. The output of both pooling ways are concatenated and then the valuable features are learned from the spatial dimension by convolutional layers. Finally, sigmod activation and element multiplication operations are performed to rescale the features. The outputs of spatial attention and channel attenion are concatenated and pass through a convolution layer to adjust the channels. Our dual attention block can be defined as Eqs (11)–(13):

$$\begin{array}{c} F_{ch}=U⊗\sigma \left(Conv1d\left(GAP\left(U\right)+STD\right)\right) \end{array}$$ (11)

$$\begin{array}{c} F_{sp}=U⊗\sigma \left(Conv7\left(GAP\left(U\right)+GMP\left(U\right)\right)\right) \end{array}$$ (12)

$$\begin{array}{c} F_{DAB}=Conv1\left(∥F_{ch},F_{sp}∥\right) \end{array}$$ (13)

where *F*_*ch*_ and *F*_*sp*_ is channel and spatial attention. *F*_*DAB*_ is our dual attention block. *Conv*7 denotes a 7 × 7 convolutional layer. *GAP* is global average pooling. *GMP* is global max pooling. STD is the standard deviation. Finally, the two branches are concatenated and a residual structure is added to stabilize the gradient propagation. In the spatial attention branch, the convolution kernel size is set as 7. In the channel attention branch, the convolution kernel of the 1-dimensional convolution is set as 3. Due to the combination of asymmetric convolution and 1D convolution, MFAB is very lightweight and also has excellent performance to efficiently extract valuable features and fade out redundant features. The reconstructed images have vivid colors and rich textures.

### 3.2 Adaptive feature aggregation block (AFAB)

**Fig 4:**
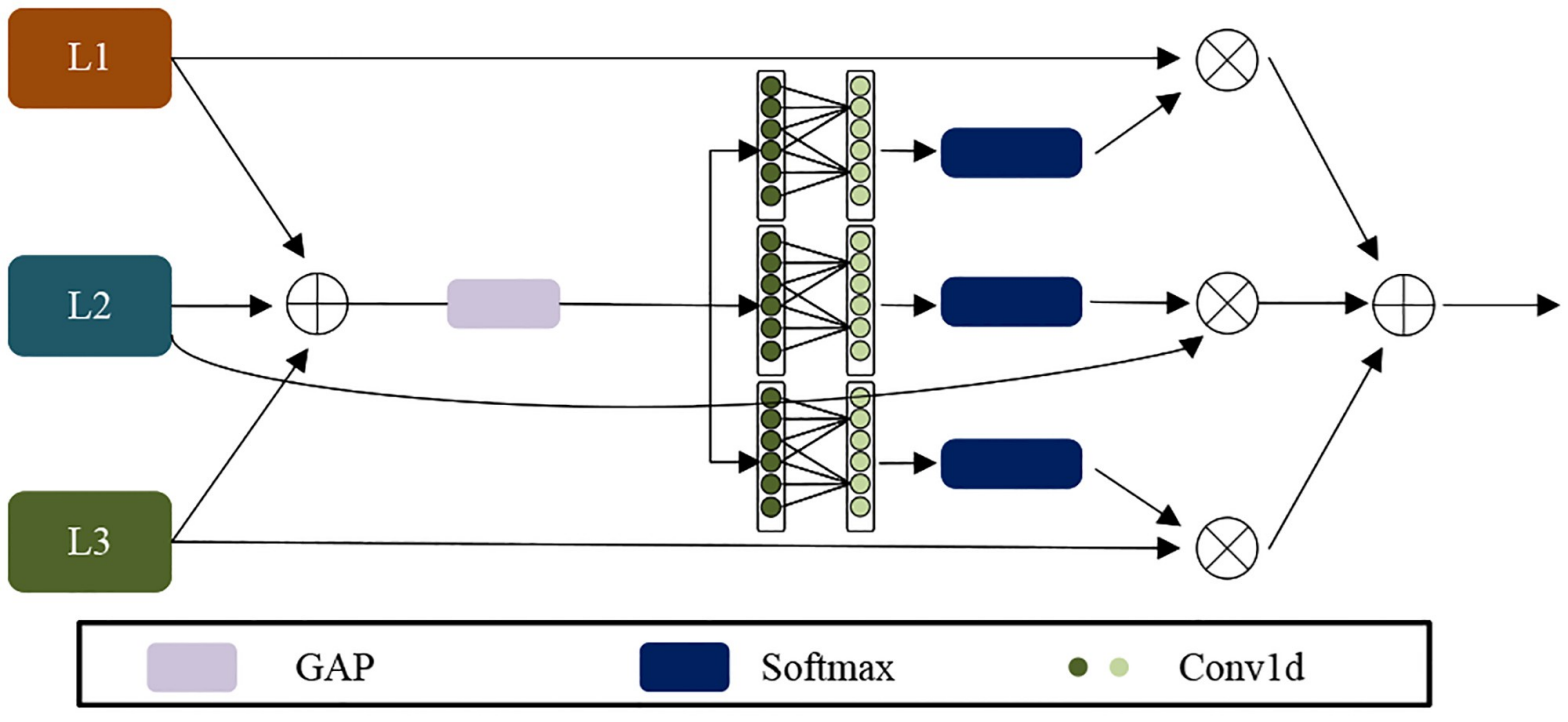
The structure of our proposed AFAB. We set the kernel of Conv1d as 3.

Existing methods often use a pyramid structure to exploit multi-scale features. The pyramid structure incorporates deep semantic information into the shallow layer through concatenation and element-wise summation operation to guide the model to learn finer features. But in practice, the inconsistency of the pyramid structure hinders the information interaction between shallow fine-grained features and deep semantic information. Liu et al. [39] proposed adaptive spatial feature fusion (ASFF) to address the effect of feature scale inconsistency on single-shot detectors. ASFF assigns weights to features to suppress feature conflicts between different scales, thus improving the scale invariance of features. Yi et al. [40] proposed an adaptive feature selection module (AFSM) for fusing neighboring scale features to improve the image dehazing effect. However, both ASFF and AFSM reduce the channel dimension so as to reduce the number of parameters, which leads to the underutilization of features. AFSM incorporates a channel attention mechanism, but the module only adaptively selects features at neighboring scales, ignoring the association of features at non-neighboring scales. We designed an AFAB, it receives features from each layer of the pyramid structure and adaptively rescales the features at each layer by the multi-branch attention structure. In addition, AFAB combines multi-branch attention structure with 1D convolution to enhance the channel information interaction and fully utilize the valuable information. As shown in Fig 4, *L*_1_ is $I_{out_{1}}$. *L*_2_ and *L*_3_ are obtained by up-sampling the outputs of $I_{out_{2}}$ and $I_{out_{3}}$. The inputs of the three branches are fused by element-wise summation. Then the fused features generate global information by global average pooling. Conv1d means 1-dimensional convolution. The softmax activation values of the three branches are multiplied by the original features on each branch and then fused by element-wise summation operation. The operation of AFAB can be defined as Eqs (14) and (15):

$$\begin{array}{c} S_{N}=L_{N}⊗\sigma \left(Conv1d\left(GAP\left(L_{1}+L_{2}+L_{3}\right)\right)\right),N=1,2,3 \end{array}$$ (14)

$$\begin{array}{c} F_{AFAB}=S_{1}+S_{2}+S_{3} \end{array}$$ (15)

where *S*_*N*_ denotes the output of the three branches of AFAB. ⊗ denotes element-wise multiplication operation. The AFAB can efficiently establish the dependencies between depth-layers semantic information and shallow fine-grained information, making full use of features at different scales. In addition, the block is lightweight and only adds a small number of parameters. The pseudo code of our proposed method is shown in Algorithm 1.

**Algorithm 1:** Efficient Adaptive Feature Aggregation Network for Low-light Image Enhancement

 **Input:** Low light ${\left\{I_{\text{input}}^{k}\right\}}_{k=1}^{N}$/Normal light sample set ${\left\{I_{gt}^{k}\right\}}_{k=1}^{N}$.

 **Output:** Enhanced image ${\left\{I_{output}^{k}\right\}}_{k=1}^{N}$.

 **Initialization:** Learning rate *η*, batch size *m*, parameter *θ*.

1 **for** *k* = 1, 2, 3 …… *N* **do**

2  *I*_1_ is extracted by Eq (1);

3  Extract the *I*_*n*_ by Eq (2), n = 2,3;

4  *F*_*ACB*_ is calculated by Eq (8);

5  *ε*_*c*_ is calculated by Eq (10), and *F*_*DAB*_ is calculated by Eq (13);

6  Extract the $I_{out_{3}}$ by Eq (4);

7  Extract the $I_{out_{n}}$ by Eq (5), n = 1,2;

8  *F*_*AFAB*_ is calculated by Eq (15);

9  Calculate by Eq (6) to update enhanced image $\left\{I_{output}^{k}\right\}$;

10  Update parameter *θ* with adam optimizer;

11  Update Learning rate *η*;

12 **end**

13 Asymmetric convolution kernels are fused by Eq (9);

14 Obtain final enhanced image $\left\{I_{output}^{k}\right\}$.

### 3.3 Loss function

We designed a loss function, which consists of two parts: Content loss and perceptual loss.

Content Loss: L2 loss tends to produce overly smooth images. The images generated by L1 loss cannot effectively suppress noises. SSIM loss measures the similarity of the output to ground-truth by brightness, contrast, and structure. The images generated by SSIM loss contain less noises and are of good quality. Therefore, we use SSIM loss as content loss. Content loss can be defined as Eqs (16) and (17):

$$\begin{array}{c} Loss_{c}=1-SSIM\left(x,y\right) \end{array}$$ (16)

$$\begin{array}{c} SSIM\left(x,y\right)=\frac{2\mu_{x}\mu_{y}+C_{1}}{\mu_{x}^{2}+\mu_{y}^{2}+C_{1}}\cdot \frac{2\sigma_{xy}+C_{2}}{\sigma_{x}^{2}+\sigma_{y}^{2}+C_{2}} \end{array}$$ (17)

where *Loss*_*c*_ is content loss. *x*, *y* are the generated image and the target image respectively. *μ*_*x*_, *μ*_*y*_ are the average values of *x* and *y* respectively. $\sigma_{x}^{2}$,$\sigma_{y}^{2}$ are the variances of *x* and *y* respectively. *σ*_*xy*_ is the covariance of *x* and *y*.*C*_1_, *C*_2_ are constants to avoid dividing by zero.

Perceptual loss: Johnson et al. [41] proposed a perceptual loss function to optimize visual effects, which is based on the VGG model. We introduce this loss function to improve the image visual quality. We use a pretrained VGG19 model, and the model weights are obtained by training on the ImageNet dataset [42]. Perceptual loss can be defined as Eq (18):

$$\begin{array}{c} Loss_{p}=\frac{1}{C_{j}H_{j}W_{j}}∥\phi_{i}\left(x\right)-\phi_{i}\left(y\right)∥ \end{array}$$ (18)

where *Loss*_*p*_ is the total loss. *C*_*j*_, *H*_*j*_, and *W*_*j*_ are the number of channels, height, and width, respectively. *ϕ*_*i*_ is the output of layer m of the VGG19 network. When we use features from different layers of VGG, the optimization effect on the model will be different, we use the output of the last convolution of the features layer to calculate the mapping differences. The two losses have a weight ratio of 1.

## 4 Experiments

### 4.1 Datasets

In experiments, we use the datasets LOL [15] and MIT5K [43]. The LOL dataset is the first paired dataset collected in real scenes, containing 485 train pairs and 15 test pairs. We follow the previous experience and extract 35 images from the training set for evaluating. The MIT5K dataset is often used by researchers in low-light image enhancement field. ln order to make a fair comparison with other methods, we use the Expert C-retouched image as the ground truth. We use 4500 pairs for training and the remaining 500 pairs for evaluating and testing. For the MIT5K dataset, we only consider image enhancement under RGB. The dataset can be made by using Lightroom software, setting the length as 500. The image sizes from the LOL dataset and the MIT5K dataset are 600 × 400 and 500 × 333, respectively.

### 4.2 Implementation details

We train our network on NVidia GTX 1080ti GPU by using the pytorch framework. The network is trained for 150 epochs. The optimizer uses the Adam optimizer, and the Adam optimizer parameters use the framework default values. We use the cosine annealing strategy and set the initial learning rate as 2 × 10^−4^. The learning rate threshold is set as 2 × 10^−6^. To enhance the robustness of the network, we use random rotation, mirroring for data augmentation. We set the batch size as 16, the patch size as 96 × 96, and set the channel as 32.

### 4.3 Comparison with typical methods

We compared some recent work on the LOL dataset, including Xu’s method [21], RetinexNet [15], GLAD [13], KinD [16], LPNet [25], Zero-DCE++ [17], RUAS [18], RAUNA [23], DRGN [24]. For the KinD method, we chose an exposure of 5 as the benchmark. Similarly, on the MIT5K dataset, we compared with some recent work including Xu’s method, White-Box [44], DPE [45], DeepUPE [22], LPNet, Zero-DCE++, RUAS, RAUNA, DRGN. The code and pre-trained models for the above methods were provided by the original authors. For Zero-DCE++, RUAS and DRGN, since the training datasets are different, we retrain the model on the two datasets to do a fair comparison. In this subsection, we compare three aspects of quantitative comparison, efficiency comparison, and visual comparison.

#### 4.3.1 Quantitative comparison

We use 2 metrics, PSNR and SSIM, for quantitative comparison. PSNR is a commonly used image objective evaluation index, the higher the PSNR value, the higher the image quality. SSIM measures the image quality in terms of luminance, contrast and structure, and the SSIM value is closer to 1, the higher the image quality. Tables 1 and 2 reflect the results of the quantitative comparison of the methods on the LOL and MIT5K datasets, where the values of PSNR and SSIM are the average of the test sets. We can see that the PSNR and SSIM of Xu’s method, Zero-DCE++, RUAS are not good on both datasets. RAUNA has good PSNR, but SSIM is not well. It can be clearly seen that our method achieved the best PSNR and SSIM on both datasets, which indicates that our method has the best quality of the images. Our method can effectively learn the mapping relationship between low luminance and normal luminance.

**Table 1:** Quantitative comparison on LOL dataset.

Method	Xu’s method	RetinexNet	GLAD	KinD	LPNet	Zero-DCE++	RUAS	RAUNA	DRGN	Ours
PSNR ↑	18.52	16.77	19.72	20.87	21.46	14.71	16.40	22.49	22.11	**22.68**
SSIM ↑	0.631	0.559	0.704	0.802	0.802	0.501	0.582	0.765	0.821	**0.829**
Param [M] ↓	-	1.23	0.93	8.49	0.15	0.01	**0.003**	1.85	4.71	0.35
FLOPs [G] ↓	-	6.79	4.37	7.44	0.77	0.24	**0.06**	28.41	25.66	1.53
Time [s] ↓	2.8715	0.3139	0.2583	0.3744	0.0179	**0.0011**	0.0041	0.0950	0.1372	0.0182

**Table 2:** Quantitative comparison on MIT5K dataset.

Method	Xu’s method	White-Box	DPE	DeepUPE	LPNet	Zero-DCE++	RUAS	RAUNA	DRGN	Ours
PSNR ↑	19.55	18.57	22.15	23.04	24.53	13.40	10.63	23.22	25.03	**25.45**
SSIM ↑	0.774	0.701	0.850	0.893	0.906	0.644	0.587	0.899	0.912	**0.924**
Param [M] ↓	-	8.56	6.67	0.75	0.15	0.01	**0.003**	1.85	4.71	0.35
FLOPs [G] ↓	-	26.10	15.36	3.46	0.77	0.24	**0.06**	28.41	25.66	1.53
Time [s] ↓	2.5122	5.9192	0.6133	0.1320	0.0169	**0.0011**	0.0040	0.0925	0.1247	0.0175

#### 4.3.2 Efficiency comparison

Efficiency is one of the important metrics for evaluating models. Existing deep learning methods are usually computationally complex and have long-running time, which greatly limits their usage scenarios. Therefore, lightweight models are gaining more and more attention. We give the number of parameters (Param), the number of floating-point operations (FLOPs) and the running time of different methods for efficiency comparison, as shown in Table 1. FLOPs are commonly used to measure the computational complexity of an algorithm. We compute FLOPs with a patch size of 96 × 96. Xu’s method is a traditional low-light enhancement method, and its running time is measured on Intel i7–10700 CPU. Other methods are measured on NVidia GTX 1080ti GPU. We can see that RUAS and Zero-DCE++ are lightweight, but the value PSNR and SSIM are not good. Since RAUNA and DRGN are not end-to-end models, their running times are limited. Our method achieves good PSNR and SSIM with a small number of parameters and running time. This shows that our method is lightweight and has a good image enhancement capability.

#### 4.3.3 Visual comparison

**Fig 5:**
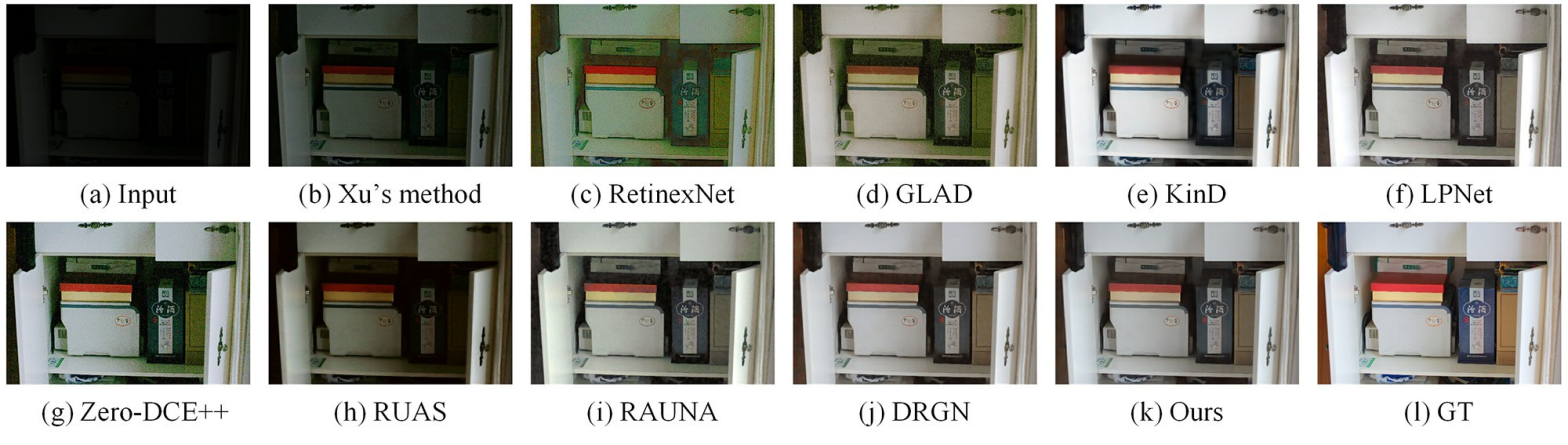
Visual results of different methods on LOL dataset for image number 55. (a) Input. (b) Xu’s method. (c) RetinexNet. (d) GLAD. (e) KinD. (f) LPNet. (g) Zero-DCE++. (h) RUAS. (i) RAUNA. (j) DRGN. (k) Ours. (l) GT.

**Fig 6:**
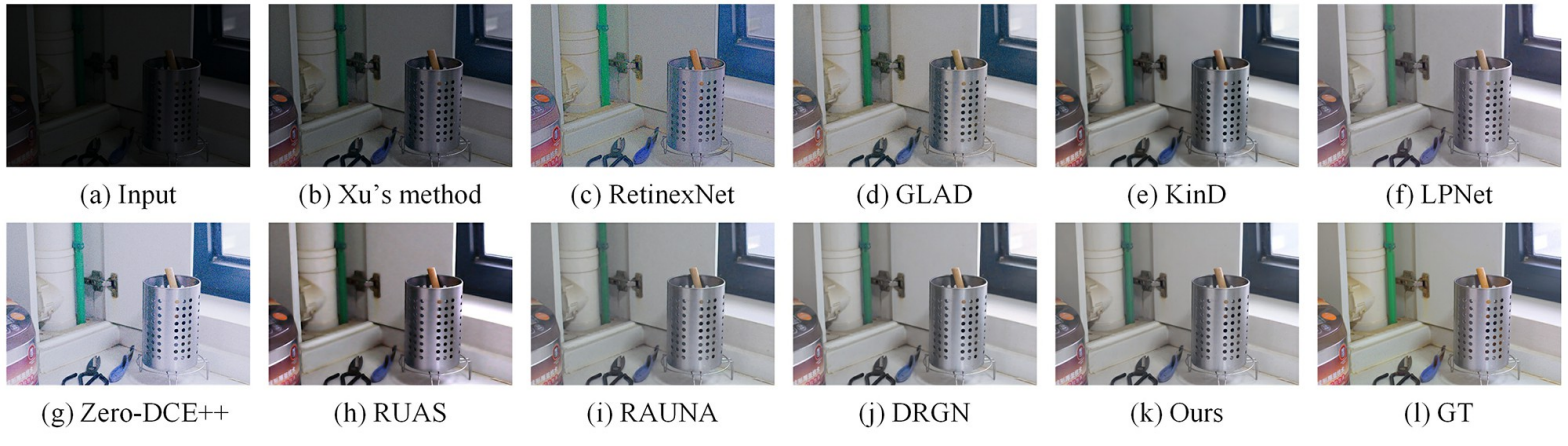
Visual results of different methods on LOL dataset for image number 111. (a) Input. (b) Xu’s method. (c) RetinexNet. (d) GLAD. (e) KinD. (f) LPNet. (g) Zero-DCE++. (h) RUAS. (i) RAUNA. (j) DRGN. (k) Ours. (l) GT.

**Fig 7:**
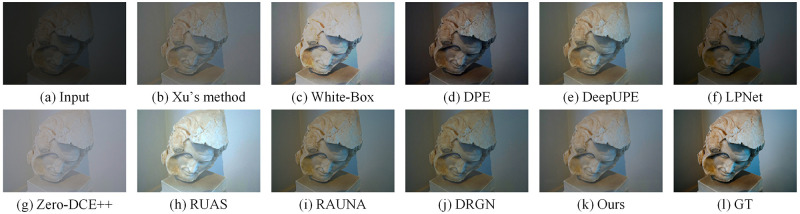
Visual results of different methods on MIT5K dataset for image number 4501. (a) Input. (b) Xu’s method. (c) White-Box. (d) DPE. (e) DeepUPE. (f) LPNet. (g) Zero-DCE++. (h) RUAS. (i) RAUNA. (j) DRGN. (k) Ours. (l) GT.

**Fig 8:**
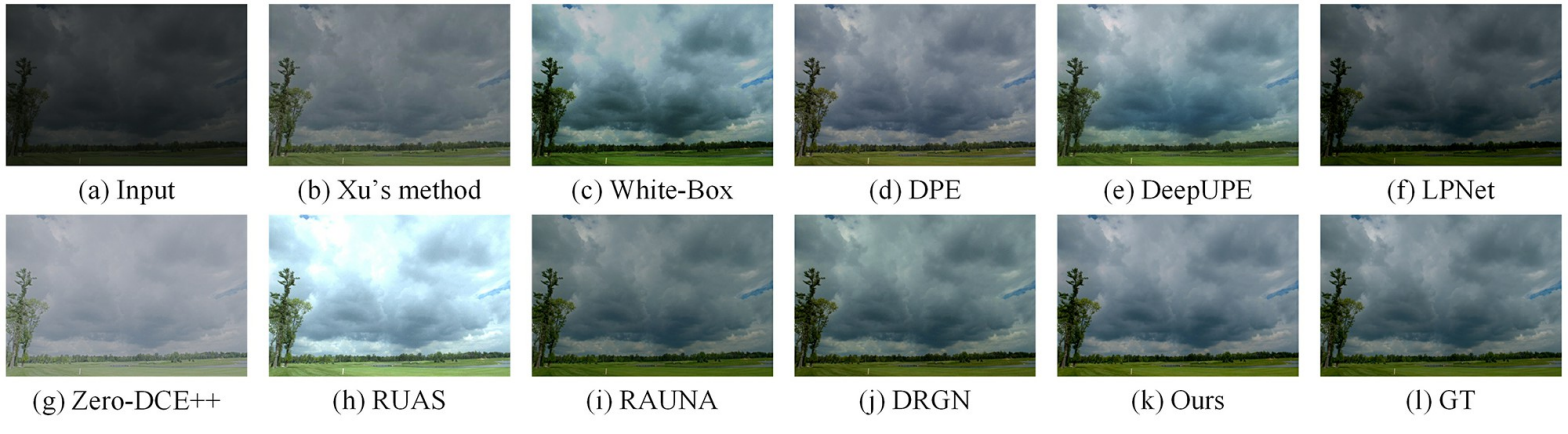
Visual results of different methods on MIT5K dataset for image number 4832. (a) Input. (b) Xu’s method. (c) White-Box. (d) DPE. (e) DeepUPE. (f) LPNet. (g) Zero-DCE++. (h) RUAS. (i) RAUNA. (j) DRGN. (k) Ours. (l) GT.

We show the visual comparison of some state-of-the-art methods on LOL and MIT5K datasets respectively. The visual comparison results on the LOL dataset are shown in Figs 5 and 6. We can see the luminance of the images reconstructed by Xu’s method are low. The images generated by GLAD and RetinexNet have a lot of noises. The images produced by KinD are too smooth and has some color distortion. LPNet and DRGN lack details. In addition, LPNet cannot handle the mirror area well. The enhanced images based on Zero-DCE++ and RUAS have a lot of noises and severe color distortion. The images recovered by RAUNA have a small amount of noises. The images generated by our method have rich colors and details while suppressing noises. The visual comparison results on the MIT5K dataset are shown in Figs 7 and 8. It can be seen that the recovered images by White-Box have overexposure. The images reconstructed by DPE and LPNet have low luminance. There are noises and color deviation in the images reconstructed by DeepUPE, RAUNA and DRGN. The enhanced images based on Zero-DCE++ and RUAS have severe distortion. The images generated by our method have better visual perception.

The above visual comparison results reflect the advantages of our method. On two datasets, the enhanced images of our method are more realistic and natural.

## 5 Ablation study

In this section, we conducted an ablation study on LOL dataset. We will investigate MFAB, the number of MFAB, pyramid structure, and AFAB. The images used in Fig 8 are from the LOL dataset.

### 5.1 Effectiveness of multi-scale feature aggregation block

We investigate the effect of MFAB, as shown in Table 3. Case 4 is the proposed method EAANet. Case 3 removes MFAB and we can see that the value of PSNR and SSIM for the model have decreased. As shown in Fig 10 (c), the visual results of the model without MFAB are obviously bad. It shows that MFAB has a positive impact on the model. MFAB contains IAB and DAB. We conduct a series of ablation studies to verify the effectiveness of each component.

**Table 3:** Study on EAANet. Pyramid, AFAB and MFAB are pyramid structure, adaptive feature aggregation block and multi-scale feature aggregation block, respectively.

**C**ase	Pyramid	AFAB	MFAB	Param [M]	FLOPs [G]	PSNR/SSIM
1	✕	✕	✓	0.38	3.53	21.87/0.803
2	✓	✕	✓	0.34	1.43	22.31/0.814
3	✓	✓	✕	0.25	1.10	21.94/0.807
4	✓	✓	✓	0.35	1.53	22.68/0.829

#### 5.1.1 Effectiveness of information aggregation block (IAB)

**Fig 9:**
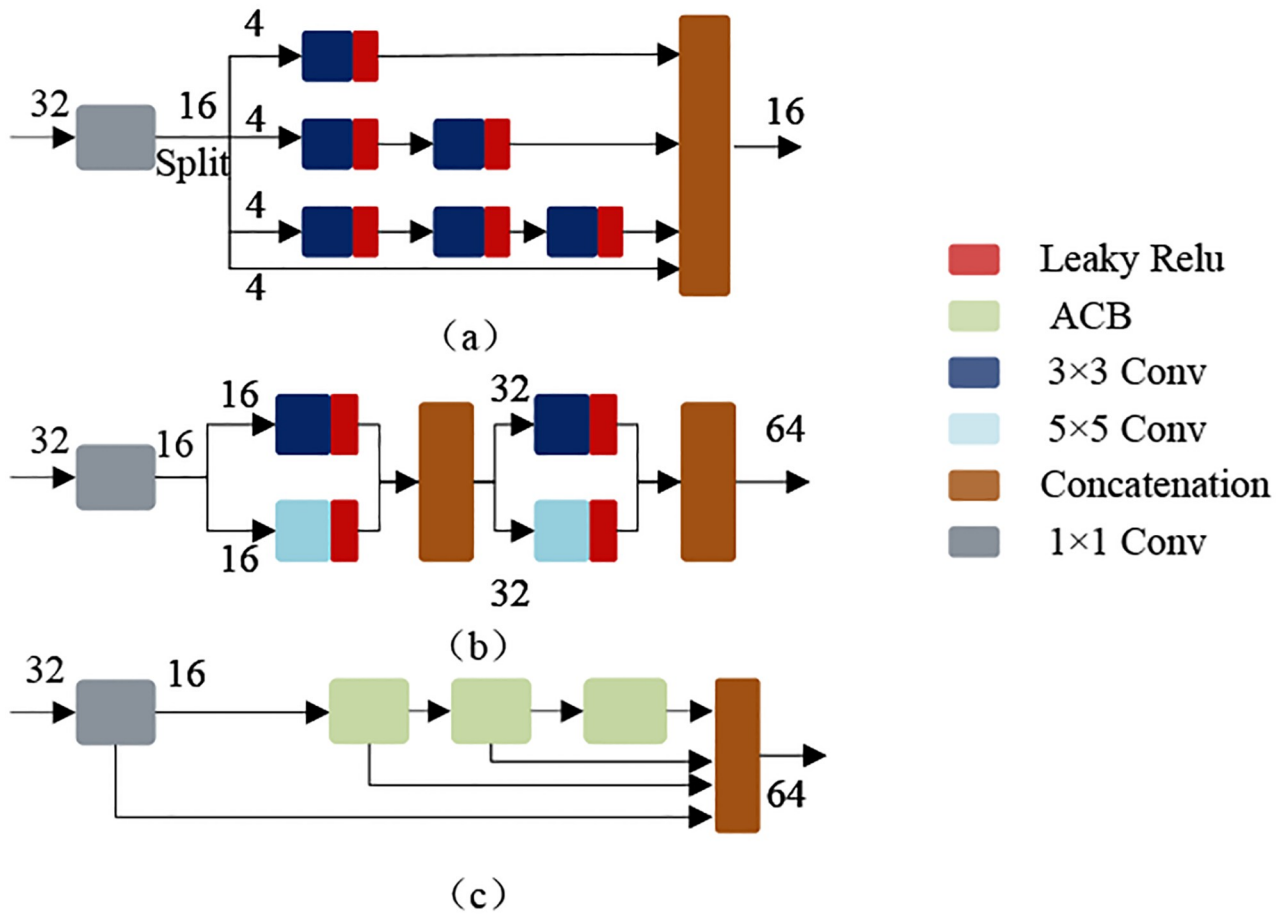
Feature extraction structure of different methods. (a) Split+Conv3. (b) Conv3+Conv5. (c) IAB.

As shown in Table 4, we use Case 3 as the benchmark data. To verify the effectiveness of IAB, we use three 3 × 3 convolutions instead of IAB. Each convolution is followed by an Leaky Relu activation. The results are shown in Case 1 of Table 4. Compared with Case 3, the PSNR and SSIM of Case 1 are significantly lower, which shows the effectiveness of IAB. In addition, we also conducted an ablation study to investigate the effect of ACB on the model, Case 2 use asymmetric convolution instead of standard convolution. We can clearly see that the PSNR and SSIM of Case 2 are higher than that of Case 1. This adequately illustrates the effectiveness of asymmetric convolution. Asymmetric convolution can extract richer features. In the inference phase, asymmetric convolution can be transformed into standard convolution to reduce the computations while maintaining the model performance. The PSNR and SSIM of Case 2 are lower than that of Case 3. This also proves the effectiveness of the aggregation structure. IAB fuses the features from different receptive fields to ensure the maximum utilization of features. We further compare with the feature extraction structure of some classical methods, as shown in Fig 9. The structure in Fig 9(a) is from LPNet. The structure in Fig 9(b) is from MSRN. We replace the IAB with the above structures for comparison experiments respectively, and the results are shown in Table 5. It can be seen that Case 3 achieves the best results. Case 1 is lightweight, but the excessive split operation limits the interaction of channels and leads to the underutilization of features. Case 2 achieves good results, but generates more parameters and computations. The above experiments illustrate that IAB is efficient.

**Table 4:** Study on Information Aggregation Block (IAB). ACB, Aggregation are asymmetric convolutional block, aggregation structure respectively.

**C**ase	ACB	Aggregation	Param [M]	FLOPs [G]	PSNR/SSIM
1	✕	✕	0.28	1.23	22.23/0.811
2	✓	✕	0.34	1.46	22.47/0.822
3	✓	✓	0.35	1.53	22.68/0.829

**Table 5:** Study on Information Aggregation Block (IAB). ACB, Aggregation are asymmetric convolutional block, aggregation structure respectively.

Case	Module	Param [M]	FLOPs [G]	PSNR/SSIM
1	Split+Conv3	0.21	0.94	22.15/0.810
2	Conv3+Conv5	0.74	3.09	22.49/0.824
3	IAB	0.35	1.53	22.68/0.829

#### 5.1.2 Effectiveness of dual attention block (DAB)

**Fig 10:**
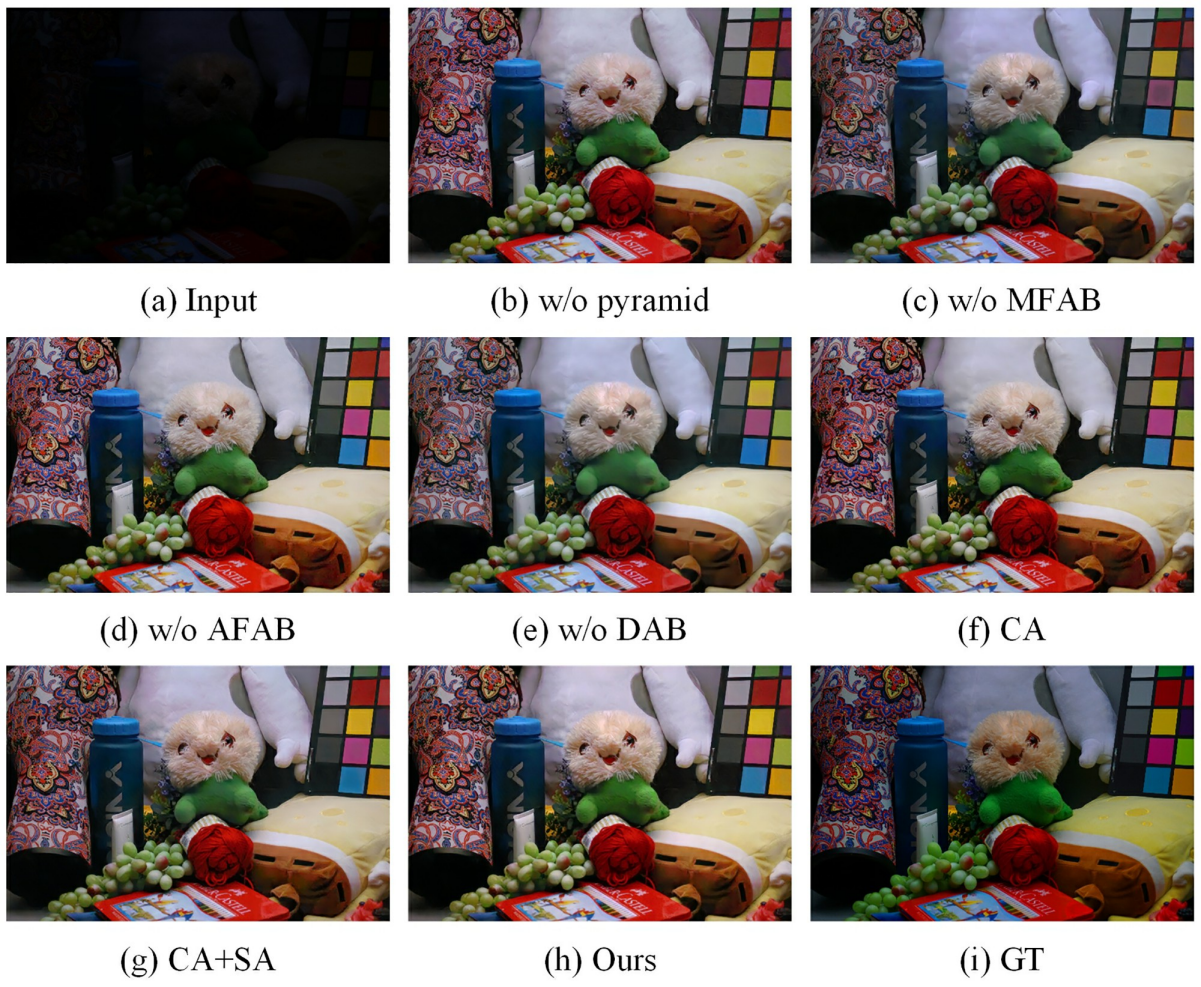
Visual results of models with different components on LOL dataset. (a) Input. (b) w\o pyramid. (c) w\o MFAB. (d) w\o AFAB. (e) w\o DAB. (f) CA. (g) CA+SA. (h) Ours. (i) GT.

We investigated the effectiveness of DAB, as shown in Table 6, and we used Case 4 as the baseline data. Compared with Case 4, the PSNR and SSIM are significantly lower in Case 1, demonstrating that the DAB has a positive effect. To further illustrate the advantages of dual attention block, we investigated the effectiveness of each component for the dual attention block. Case 2 uses only channel attention (CA). Case 3 adds spatial attention (SA) to CA. We can see that both CA and SA can improve the model. Case 4 is the proposed DAB. We can see that Case 4 has the best performance, which proves the effectiveness of STD. As shown in Fig 10(h) has the best visual effect.

**Table 6:** Study on Dual Attention Block (DAB). CA,SA,STD are channel attention, spatial attention, and standard deviation respectively.

**C**ase	CA	SA	STD	Param [M]	FLOPs [G]	PSNR/SSIM
1	✕	✕	✕	0.34	1.48	22.13/0.807
2	✓	✕	✕	0.34	1.48	22.35/0.816
3	✓	✓	✕	0.35	1.53	22.49/0.816
4	✓	✓	✓	0.35	1.53	22.68/0.829

The above study verifies the effectiveness of IAB and DAB. IAB can extract features efficiently, while DAB can reduce redundant features.

### 5.2 Study on the number of multi-scale feature aggregation blocks

In this part, we explored the effect of the number of multi-scale feature aggregation blocks (MFABs). In general, deeper network has more parameters and stronger fitting ability. We provided the results of PSNR, SSIM, parameters, computations and the running time by using different numbers of MFABs, as shown in Table 7. When the number of MFABs gradually increases, the model can obtain better PSNR and SSIM, but the parameters, computations and running time of the model also rise. When the number of MFABs is more than 4, the rising trend of PSNR becomes slow and SSIM appears to decrease, while the parameters and computations of the model increase greatly. In terms of lightweight and efficiency, we set the number of MFABs as 4.

**Table 7:** Study on the number of Multi-scale Feature Aggregation Blocks (MFABs).

Number	PSNR/SSIM	Param [M]	FLOPs [G]	Time [s]
2	22.23/0.806	0.24	1.05	0.0140
3	22.37/0.811	0.30	1.29	0.0159
4	22.68/0.829	0.35	1.53	0.0182
5	22.71/0.824	0.41	1.75	0.0198

### 5.3 Effectiveness of network structure

In this part, we performed a series of ablation experiments on network structure, pyramid structure and AFAB. We explored the impact of the pyramid structure, as shown in Table 3. Case 1 removes the pyramid structure and AFAB, retains only the shallow branches, and doubles the number of channels to equal the number of parameters in Case 4 for a fair comparison. We can see that the PSNR and SSIM of Case 1 are significantly lower due to the lack of a pyramid structure. As shown in Fig 10, the visual results of the model without the pyramid structure are significantly worse. The experimental and visual results show that the pyramid structure is effective.

We investigated the effect of AFAB and the results are shown in Table 3. Case 2 removes AFAB, and it can be seen that the PSNR and SSIM of Case 2 are significantly lower. We give the corresponding visual effects, as shown in Fig 10, the images generated by the model using AFAB have better contrast and color. AFAB solves the inconsistency of the pyramid and enables the deep semantic information to better guide the shallow fine-grained information to learn finer textures and colors. To illustrate the advantages of the proposed AFAB, we further compare it with ASFF [39] and AFSM [40]. Since AFSM only performs adaptive fusion of neighboring scale features, we improved it to receive features from each layer of the pyramid. As shown in Table 8, we can see that AFAB obtains the best value of PSNR and SSIM. This indicates that AFAB can better adaptively fuse the features of different scales.

**Table 8:** Study on Adaptive Feature Aggregation Block (AFAB).

Case	Module	Param [M]	FLOPs [G]	PSNR/SSIM
1	ASFF	0.35	1.53	22.43/0.823
2	AFSM	0.36	1.55	22.31/0.820
3	AFAB	0.35	1.53	22.68/0.829

In summary, all parts of the model are effective, and our model achieves good performance with the synergy of each component.

## 6 Conclusions

In this paper, we design an efficient adaptive feature aggregation network for low-light image enhancement. We propose two important modules, MFAB and AFAB, to construct the proposed network. MFAB efficiently extracts features using asymmetric convolution and a dual attention mechanism. By using MFAB, the reconstructed image has rich texture and the noises is effectively suppressed. AFAB combines one-dimensional convolution to efficiently rescale the features of each branch, it overcomes the inconsistency of the pyramid structure and improves the luminance, color and texture deviation of the enhanced images. Extensive experiments and ablation studies have shown that the proposed method has significant advantages over state-of-the-art methods. Meanwhile, the method has a quick running time, which has great potential for assisting advanced vision tasks or applying on mobile. In the future, we will further validate the proposed method in more image restoration tasks, such as image de-snowing and de-raining task.

## Supporting information

S1 File (ZIP)

S2 File (RAR)
